# Adherence to clinical practice guidelines amongst adolescents with buccal fixed orthodontic appliances in northeast Netherlands: a cross-sectional study

**DOI:** 10.1093/ejo/cjaf041

**Published:** 2025-06-15

**Authors:** Sebastiaan P van Doornik, Scott Lietmeijer, Yijin Ren, David J Manton, Pieter U Dijkstra, Anne Marie Kuijpers-Jagtman

**Affiliations:** Department of Orthodontics, University Medical Center Groningen, Hanzeplein 1, 9713 GZ, Groningen, The Netherlands; Center for Dentistry and Oral Hygiene, University of Groningen, University Medical Center Groningen, Antonius Deusinglaan 1, FB 21, 9713 AV Groningen, The Netherlands; Department of Orthodontics, University Medical Center Groningen, Hanzeplein 1, 9713 GZ, Groningen, The Netherlands; Center for Dentistry and Oral Hygiene, University of Groningen, University Medical Center Groningen, Antonius Deusinglaan 1, FB 21, 9713 AV Groningen, The Netherlands; Academic Center for Dentistry (ACTA), Gustav Mahlerlaan, 3004 Amsterdam, The Netherlands; Department of Rehabilitation, University Medical Center Groningen, University of Groningen, Hanzeplein 1, 9713 GZ Groningen, The Netherlands; Department of Oral and Maxillofacial Surgery, University Medical Center Groningen, University of Groningen, Hanzeplein 1, 9713 GZ Groningen, The Netherlands; Sirindhorn School of Prosthetics and Orthotics, Faculty of Medicine Siriraj Hospital, Mahidol University, 14 Arun Amarin Rd, Bangkok 10700, Thailand; Department of Orthodontics, University Medical Center Groningen, Hanzeplein 1, 9713 GZ, Groningen, The Netherlands; Department of Orthodontics and Dentofacial Orthopedics, School of Dental Medicine/Medical Faculty, University of Bern, Hochschulstrasse 4, 3012, Bern, Switzerland; Faculty of Dentistry, Universitas Indonesia, Campus Salemba, Jalan Salemba Raya No. 4, Jakarta 10430, Indonesia

**Keywords:** orthodontics, orthodontic treatment, caries prevention, adolescent oral health, buccal fixed appliances, adherence, clinical practice guideline

## Abstract

**Background:**

Adolescents undergoing orthodontic fixed appliance treatment face an increased risk of dental caries and developing white spot lesions (WSLs) due to challenges in maintaining effective oral hygiene. Preventive measures, including adherence to clinical guidelines, are important to reduce these risks.

**Objective:**

To analyse adherence to clinical practice guidelines (CPGs) amongst patients undergoing orthodontic treatment with buccal fixed appliances in the northeast of the Netherlands.

**Methods:**

A survey was presented to 539 adolescents aged 12–17 undergoing buccal fixed appliance treatment. Participants were recruited from ten orthodontic practices. The survey assessed adherence to the six recommendations of the Dutch CPGs. An adherence sum score (range 0 to 6) was calculated. Descriptive statistics and linear regression analyses (1000 bootstrap samples) were performed to analyse the relationships between participants’ characteristics and adherence sum scores.

**Results:**

In total, 485 adolescents started the survey, of whom 393 (72.9%) could be included (57% female; 48.9% aged 13 or 14). The median adherence sum score was 5 (IQR 4, 5), and 22.6% (n = 89) had an adherence sum score of 6. Males had a lower adherence sum score than females (−0.442, 95% CI: −0.979, −0.234). Older participants had a lower adherence sum score than younger participants (−0.066, 95%CI: −0.136, 0.002) per year of age. Higher educated participants had a lower adherence sum score than participants with lower education (−0.534, 95%CI: −0.953, −0.096).

**Limitations:**

Self-reported data may introduce information bias as participants may give socially desirable answers.

**Conclusions:**

Adherence to the CPGs amongst adolescents with buccal fixed orthodontic appliances is suboptimal, particularly in boys and older adolescents. Tailored educational interventions may address these gaps.

## Introduction

Orthodontic treatment involving buccal fixed appliances poses an increased risk of caries lesion development due to challenges in maintaining effective oral hygiene [1–3]. Brackets on the buccal surfaces of the teeth impede cleaning, resulting in plaque accumulation, increased risk of enamel demineralisation, and the formation of white-spot lesions (WSLs) [1, 3–6]. WSLs are not only an aesthetic concern but may also compromise treatment outcomes because of early discontinuation of the orthodontic treatment and an increased risk of caries lesion progression [2, 6–8].

The prevalence of WSLs in orthodontic patients ranges widely from 34% to 97%, emphasising the considerable impact of oral hygiene practices and dietary habits [5–8]. Adolescents undergoing orthodontic treatment with buccal fixed appliances are particularly susceptible to negative outcomes due to reduced motivation for oral hygiene and frequent consumption of cariogenic food [4, 8]. Inadequate adherence of caries preventive measures amongst adolescents underscores the necessity of assessing attitudes and practices related to oral health in this population [4].

Optimal oral hygiene relies on professional instructions, appropriate hygiene tools, and consistent patient adherence [3, 5, 8, 9]. Emphasising a healthy diet, limiting the consumption of cariogenic food, maintaining orthodontic appliances, and scheduling regular dental check-ups are crucial for both oral hygiene maintenance and caries risk management [5, 8–11]. Neglecting these recommendations increases the caries risk [5, 10, 11]. Improved oral health knowledge is associated with better oral health practices and increased awareness of patients undergoing buccal fixed orthodontic therapy [4].

In the Netherlands, the clinical practice guidelines (CPGs) ‘’Advice on Caries Prevention (ACP)’’, introduced in 2011, outline three main components for all dental professionals: dental hygiene, fluoride use, and dietary recommendations [12]. This comprehensive ACP guideline was later incorporated into the White Spot Lesions CPG for orthodontists (2022), focussing on preventing these lesions in orthodontic patients [5]. The implementation of these CPGs in orthodontic practices is also a regulatory requirement for the five year orthodontic accreditation and re-registration of an orthodontic specialist.

The northeast of the Netherlands exhibits a lower socioeconomic status (SES) compared to other areas, leading to disparities in oral health [10, 11]. The prevalence of caries is increased amongst low-SES households due to the higher consumption of cariogenic and processed foods compared to higher-SES households [10, 11]. Given the high percentage of adolescents in orthodontic treatment and the unique challenges posed by lower SES in the northeast, it is important to understand how well these adolescents adhere to the CPGs. This insight may help to develop tailored interventions. It is hypothesised that any deviation from the recommendations of the CPGs may increase the patient’s risk of WSL development and that certain patient characteristics are associated with guideline adherence.

This study aimed to analyse the adherence to the CPGs amongst adolescents (aged 12–17 years) undergoing orthodontic treatment with buccal fixed appliances in the northeast of the Netherlands. Additionally, this study analysed the associations of participant characteristics with adherence to these guidelines, exploring oral hygiene habits amongst adolescents and their associations with factors such as sex, SES, and (parental) education levels.

## Methods

### Study design, ethical considerations and informed consent

This study was a cross-sectional survey with an analytical purpose to gain insight into the participant characteristics associated with adherence to the CPGs [5, 12]. The Medical Ethics Review Board of the University Medical Centre of Groningen (METc Groningen, METc 2023/014) evaluated the survey and determined it did not qualify as clinical research involving human subjects under the Medical Research Involving Human Subjects Act (WMO). Participants received an explanation of the study through a plain language statement incorporated in the survey. Written informed consent was obtained from both participants and their parents or guardians.

### Survey development and validation

The survey assessed participant characteristics (7 questions), including age, sex, participant education level, mother’s education level (SES household estimation), and postal code to estimate the SES and adherence to the CPGs (6 questions) [5, 12] (supplementary file 1). Participants were considered to have adhered to the CPGs if they (1) brushed their teeth twice-per-day, (2) brushed for two minutes, (3) used fluoride toothpaste, (4) used interdental cleaning, (5) visited an external dental clinic at least once-per-year, and (6) their food and fluid intake frequency did not exceed seven times-per-day. Participants received one point for each item adhered to, resulting in an adherence sum score ranging from 0 to 6 [5, 12]. The survey took approximately 10 minutes to complete.

The survey was reviewed regarding content validity and accuracy by experienced faculty members of the Departments of Orthodontics and Cariology of the UMCG. This survey was piloted amongst 44 patients undergoing orthodontic treatment at the UMCG, Department of Orthodontics, to assess content validity and identify areas of confusion and improvements. After adaptations based on reviewer and patient comments, the survey had sufficient internal consistency (Cronbach’s Alpha = 0.716).

### Participant recruitment and data collection

The inclusion criteria were age between 12 and 17 years and currently undergoing treatment with full fixed appliances in both arches with buccal brackets from first permanent molar to first permanent molar for a minimum of three months. Exclusion criteria were patients with cleft lip and/or palate or other craniofacial anomalies, intellectual disabilities requiring special treatment, participation in the pilot study, and individuals or parents lacking sufficient proficiency in the Dutch language to provide informed consent or to understand the survey. Participants were recruited from orthodontic practices affiliated with the Orthodontics Research Network Northeast Netherlands. The researchers (SvD, SL, RK) visited these practices, identified eligible patients and provided them with information about the study. SvD was responsible for the training process and calibrated the investigators to ensure consistency in data collection, both during the recruitment and the data collection phase. Upon approach, patients were asked if they would like to participate in a survey, followed by questions addressing the inclusion and exclusion criteria, including their age and whether they were wearing buccal fixed appliances. Practitioners and caregivers were not involved in participant recruitment.

### Data security and management

The survey was distributed using the Research Electronic Data Capture programe (REDCap, Indiana University Pervasive Technology Institute, Indiana, USA) hosted on the UMCG network [13, 14]. Participants accessed the survey via a unique and secured link provided by the researchers on-site. Survey responses were encrypted and securely stored. The data management process adhered to the General Data Protection Regulation (GDPR) and was only accessible to the researchers.

### Sample size calculation

The sample size calculation was conducted in G*Power (Heinrich-Heine-Universität Düsseldorf, Düsseldorf, Germany). Settings for the calculations were an effect size of 0.15 for 5 predictor variables for a multivariable regression analysis. Alpha was set at 0.05, and the power was set at 0.80. The result of the calculation was a sample size of 55 participants per predictor variable, totalling 275 participants. The non-response rate was estimated to be approximately 50%. To ensure a sufficient sample size, the aim was to invite 550 potential participants. The non-response rate estimate was based on previously conducted surveys [15].

### Data analysis

Descriptive statistics summarised participant characteristics such as age, sex, education of participant and mother, postal code, and survey responses. The education of the participant and mother was categorised based on the Dutch education system as:

- low (primary school and lower secondary vocational education),
- middle (senior general secondary education and secondary vocational education),
- high (pre-university education, university of applied sciences and university).

Treatment duration with buccal fixed appliances was categorised into 0 to less than 3 months, 3 to less than 6 months, 6 to less than 12 months, and 12 months or longer.

Univariate analyses were applied to explore associations between participant characteristics and adherence sum scores. Sex and SES were analysed using the Mann-Whitney U test. Education and treatment duration were analysed using the Kruskal-Wallis Test. The association between age and adherence sum score was analysed using Pearson’s coefficient. A multivariable linear regression model included all participant characteristics significantly associated with the adherence sum score. Due to unmet regression assumptions, bias-corrected bootstrapping (1000 samples) was applied. Additionally, interaction effects were explored.

## Results

### Sample

**Figure 1.**
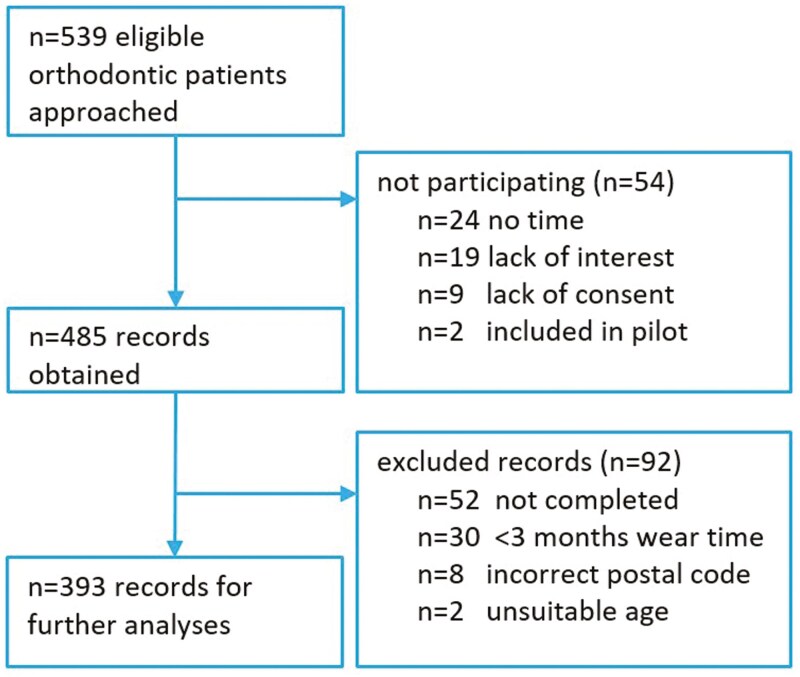
Study Flowchart.

Ten specialist orthodontic practices participated in this study and were visited between February and April 2023 (Supplementary file 2). In total, 539 participants were approached, of which 485 (90.0%) participated. Furthermore, 92 surveys were excluded due to incompletion, incorrect postal codes, unsuitable age, and not meeting the minimum wear time, resulting in 393 (72.9%) complete records (Fig. 1).

### Participant characteristics

A majority were girls (n = 225, 57.3%), and the most frequent ages were 13 (n = 108, 27.5%) and 14 years (n = 84, 21.4%) (Table 1). Most participants followed higher education (n = 179, 45.6%) or middle education (n = 175, 44.6%). Most mothers had completed higher education (n = 170, 43.3%), where a quarter of the participants were unsure of their mothers’ educational level. Most participants had been in treatment for over 12 months (n = 225, 57.3%).

**Table 1.** Participant characteristics.

Participant characteristics Total n = 393	Frequency N (%)		Median (IQR)	*P*-value	Statistical test
Sex*				<0.001	MWU
male	157	(39.9%)	5 (4, 5)		
female	225	(57.3%)	5 (4, 6)		
different	5	(1.3%)	5 (3.5, 5.5)		
prefer not to disclose	6	(1.5%)	5 (4, 5.3)		
**Age in years**				0.031	KW
12	64	(16.3%)	5 (4, 6)		
13	108	(27.5%)	5 (4, 6)		
14	84	(21.4%)	5 (4, 5)		
15	56	(14.2%)	5 (4, 5)		
16	53	(13.5%)	5 (4, 5)		
17	28	(7.1%)	5 (4, 5)		
Current education level participant**				0.022	KW
low education	19	(4.8%)	6 (4, 6)		
middle education	175	(44.6%)	5 (4, 6)		
high education	179	(45.6%)	5 (4, 5)		
none	3	(0.8%)			
different	17	(4.3%)			
Completed education level mother**				0.218	KW
low education	2	(0.5%)			
middle education	115	(29.3%)	5 (4, 6)		
high education	170	(43.3%)	5 (4, 5.3)		
none	7	(1.8%)	4 (2.5, 5)		
don’t know	97	(24.7%)			
different	2	(0.5%)			
**Months in orthodontic treatment**				0.869	KW
3 to < 6 months	57	(14.5%)	5 (4, 6)		
6 to <12 months	111	(28.2%)	5 (4, 5)		
≥ 12 months	225	(57.3%)	5 (4, 5)		
Adherence Sum score***			5 (4, 5)		
1	4	(1.0%)			
2	9	(2.3%)			
3	40	(10.2%)			
4	106	(27.0%)			
5	145	(36.9%)			
6	89	(22.6%)			

Statistical tests were applied to analyse differences between the groups with regard to adherence sum score MWU; Mann Whitney U test, KW: Kruskal- Wallis test, * comparison between boys and girls, ** comparison between low, middle and high education, ***total points for participants who met the CPGs.

### Daily oral care habits and calculation of the adherence sum score

**Figure 2.**
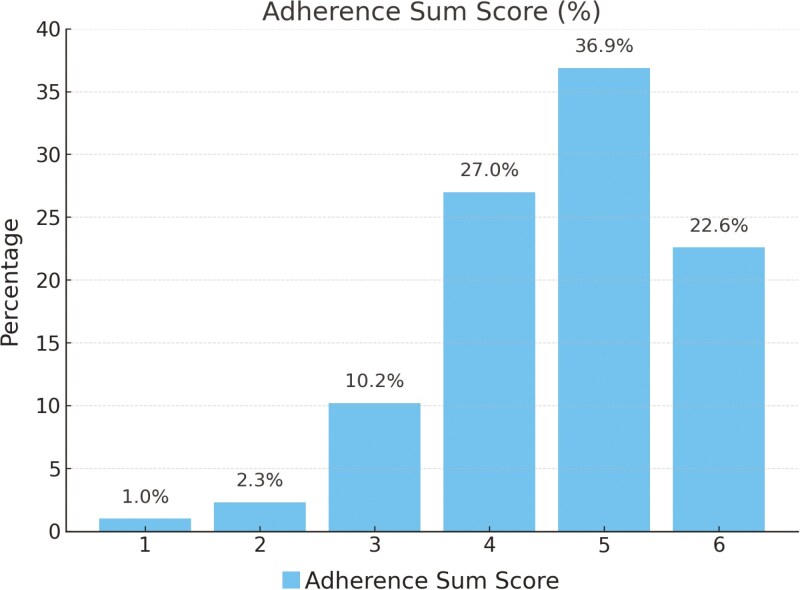
Distribution Adherence Sum Score.

A majority (n = 348, 88.6%) reported brushing their teeth at least twice daily (item 1 adherence sum score) (Table 2). Additionally, 88.5% (n = 348) adhered to the recommended brushing duration of at least two minutes (item 2 adherence sum score). The frequency of use of a powered or manual toothbrush was 35.6% and 35.1% of the participants, respectively. Amongst participants, 44.0% (n = 173) used fluoride-containing toothpaste (item 3 adherence sum score), while 46.6% (n = 188) were unaware if their toothpaste contained fluoride. Participants using non-fluoridated toothpaste were found not to adhere to item 3. Almost all participants (n = 388, 98.7%) used a toothbrush, with a considerable proportion also utilising interdental brushes (n = 207, 52.7%) and fluoride mouth rinses (n = 114, 29.0%) (Table 3). The use of additional dental cleaning products beyond toothbrushing (manual or electric) indicated adherence to item 4. Furthermore, most respondents (n = 336, 85.5%) had visited their dentist or oral hygienist in the past year during the orthodontic treatment (item 5 adherence sum score). In terms of dietary habits, 80.9% (n = 318) reported consuming foods or drinks up to 6 to 7 times daily (item 6 adherence sum score). In total 22.6% (n = 89) of the participants adhered to all six recommendations (Fig. 2).

**Table 2.** Oral care habits of the participants (N = 393).

	n %	
*Tooth Brushing frequency (per day)*		
< 1	3	(0.8%)
1	42	(10.7%)
2	304	(77.4%)
3	41	(10.4%)
4	3	(0.8%)
*Brushing time per session (minutes)*		
1	36	(9.2%)
2	219	(55.7%)
3	83	(21.1%)
4	27	(6.9%)
> 4	19	(4.8%)
don’t know	9	(2.3%)
*Mode of toothbrushing*		
manual	140	(35.6%)
powered	138	(35.1%)
both	115	(29.3%)
*Toothpaste type*		
with fluoride	173	(44.0%)
without fluoride	14	(3.6%)
both	23	(5.9%)
don’t know	183	(46.6%)
*Routine dental check-ups*		
no	37	(9.4%)
yes	336	(85.5%)
don’t know	20	(5.1%)
*Food and beverage consumption (times per day)*		
< 4	19	(4.8%)
4–5	138	(35.1%)
6–7	161	(41.0%)
8–9	60	(15.3%)
10–11	12	(3.1%)
> 11	3	(0.8%)

**Table 3.** Use of dental cleaning products in 393 participants.

	Frequency n(%)			
Use of dental tools	No		Yes	
Tooth brush	5	1.3%	388	98.7%
Interdental brushes	186	47.3%	207	52.7%
Toothpicks	284	72.3%	109	27.7%
Dental floss	273	94.9%	20	5.1%
Fluoride mouthrinse	279	71.0%	114	29.0%

In the univariate analyses, the adherence sum score was significantly associated with sex, boys scoring lower than girls; with age, older participants scored slightly lower than younger participants, and with education levels, participants with higher levels of education were associated with lower scores compared to those with lower levels of education (Table 1).

### Regression analysis

In the regression analyses, the association of sex and participant education level remained statistically significant, whilst the association of age was borderline significant (p = 0.082; Table 4). The explained variance of the model was 7.3%. The interaction between age and sex was not significantly associated with the adherence sum score.

**Table 4.** Results from linear regression analyses.

Model	Coefficients^*^	SE	*P*-value	95% CI B	
	B			Lower Bound	Upper Bound
(Constant)	5.405	0.216	<0.001	4.962	5.832
Higher education	−0.534	0.233	0.021	−0.953	−0.096
Middle education	−0.404	0.236	0.079	−0.832	0.025
Sex (boy)	−0.442	0.112	<0.001	−0.679	−0.235
Age centred (at 12 years)	−0.066	0.037	0.082	−0.136	0.002

^*^Dependent Variable: Adherence sum score. The reference category is a girl of 12 years following lower education. The explained variance of the results was 7.3%.

## Discussion

The present study aimed to evaluate adherence to the CPGs amongst adolescents undergoing orthodontic treatment with buccal fixed appliances in the northeast of the Netherlands using a cross-sectional survey. Patients who had been treated with buccal fixed appliances in both arches for at least three months were included, as these appliances are commonly prescribed in the Netherlands and are associated with an elevated risk of developing WSLs [2, 11]. Consequently, this patient group was classified as high-risk for caries lesion development.

The results revealed that only 22.6% of the adolescents adhered to the six CPG recommended oral hygiene practices. As hypothesised, each deviation from recommendations of the CPGs increases the patient’s risk for adverse oral health effects such as WSL development. Insufficient adherence to oral hygiene practices may contribute to inadequate plaque control, prolonged exposure to cariogenic biofilms, and subsequent enamel demineralisation, all known adverse effects of buccal fixed orthodontic treatment [1–4, 6, 8].

The present study also examined the influence of various participant characteristics, including age, sex, SES, and education level, as these factors have been reported to affect oral health behaviours [10, 16, 17]. Findings revealed that sex, age, and participant education were significantly associated with CPG adherence. Females demonstrated higher adherence than males, younger adolescents adhered more closely to the CPGs than older ones, and participants in lower education levels exhibited better adherence than those in higher education levels. The finding of better adherence amongst females is consistent with the findings of earlier studies [18, 19].

### Orthodontic treatment and socioeconomic context

Although earlier studies found differences in the prevalence of orthodontic treatment related to SES, we did not find any differences [19, 20]. These differences could be because, in the Netherlands, the prevalence of orthodontic treatment is high, ranging from 53% to 80%, largely due to a unique insurance policy that enhances accessibility and keeps costs relatively low for the patient [7, 11]. As in previous studies, postal code and education of the mother were used as surrogate indicators of SES [10, 11, 21]. Interestingly, participants at lower educational levels demonstrated slightly better adherence to the guidelines. While 25% of participants were unaware of their mothers’ educational background, the sample size provided sufficient statistical power to detect differences in the CPGs adherence, but this variable did not yield statistically significant results.

### Oral health habits

At the onset of orthodontic treatment, patients often improve their oral hygiene routines and dietary habits [22]. However, after three months of orthodontic treatment, dietary habits are similar to those before treatment [23]. To reduce the potential confounding effects of early treatment, we excluded patients who had buccal fixed appliances for less than three months. In total, 88.5% of participants reported brushing their teeth at least twice daily, which aligns with the recommendations of the CPGs. This adherence rate is higher than reported for the general Dutch adolescent population, where only 68% in low SES groups and 80% in high SES groups brush twice daily [11]. Our findings suggest that orthodontic patients may be more conscientious regarding oral hygiene compared to their peers not treated with buccal fixed appliances.

Awareness of fluoride use was suboptimal, with many participants unaware of whether their toothpaste contained fluoride. Given fluoride’s pivotal role in reducing caries risk, this lack of awareness poses a considerable risk [2, 6, 24]. However, it is important to note that young adults who do not buy their own toothpaste may not know about the importance of fluoride use. In the present study, the few participants who reported using non-fluoride toothpaste did not adhere to the CPGs, although this could represent an underestimation.

Similarly, adherence to interdental cleaning practices was suboptimal, with only 52.7% of participants using interdental brushes, however, in the general population, use is even lower, with approximately 25% using interdental cleaning tools daily [11].

Dietary habits also played a role in adherence, with 81% of participants adhering to the recommended limit of seven or fewer daily eating or drinking occasions. This percentage is higher than in the general adolescent population, where 67% of low SES and 80% of high SES adolescents adhere to the recommended daily limit [11].

### Study limitations

First, the self-reported nature of the data may introduce information bias, such as giving socially desirable answers, which might have resulted in an overestimation of adherence, however, the present results are consistent with previous research [11]. In Dutch orthodontic practices, the use of the CPGs is a regulatory requirement. Adherence to them is a key component of the five yearly re-registration process for orthodontic specialists in the Netherlands, supporting alignment with professional standards [25]. The present study did not investigate the implementation of the guideline; therefore, examining how the CPGs were presented to participants was beyond the scope of this research. The regional focus of the study may limit the generalizability of the results to populations with different characteristics. The explained variance of the results was 7.3%, which shows that the associated factors account for only a small part of the variability in adherence. Another limitation is the potential for participants to misunderstand certain survey questions; therefore, the researcher was always present in person for further clarification. This presence is particularly relevant for questions regarding recommended maximum food and fluid intake, mothers’ education, and fluoride in toothpaste. The present study has limitations associated with being a survey-based study; however, a large sample size was included within a real-world setting.

### Future directions

The findings of the present study emphasise the need for tailored educational interventions that address the specific needs and interests of young orthodontic patients, more specifically, male, older and higher educated participants. A stronger focus on interdental cleaning and fluoride use should be considered, where instructions need to be repeated more frequently to reach a better understanding of these topics. Future research could investigate the effectiveness of different intervention strategies in enhancing adherence to the CPGs for oral health care management during orthodontic treatment. Longitudinal studies are needed to evaluate changes in adherence over time and assess the long-term impact of these interventions on oral health outcomes and the development of WSLs.

## Conclusion

In conclusion, whilst most adolescents undergoing buccal fixed orthodontic treatment exhibited adequate oral hygiene practices, insufficient adherence remained an issue, particularly regarding fluoride use and interdental cleaning. The present study highlights the complexity of oral health care management in orthodontic patients. Factors such as age, sex and participant education levels were statistically significantly associated with adherence to the CPGs, whereas SES and maternal education did not play a significant role. Given the increased risk of WSLs and other oral health complications associated with orthodontic appliances, it is important to address the lack of adherence through targeted education and intervention strategies during and after treatment.

## Supplementary Material

cjaf041_suppl_Supplementary_Files_1

cjaf041_suppl_Supplementary_Files_2

## Data Availability

All data analysed during this study are included in this published article. Primary data are available upon reasonable request to the corresponding author.
