# Supplementary material for: Adherence to clinical practice guidelines amongst adolescents with buccal fixed orthodontic appliances in northeast Netherlands: a cross-sectional study
Source: Eur J Orthod. 2025 Jun 15;47(4):cjaf041. doi: 10.1093/ejo/cjaf041 (PMC12167228; doi:10.1093/ejo/cjaf041)
Supplement: cjaf041_suppl_Supplementary_Files_1 [file cjaf041_suppl_supplementary_files_1.docx]

Supplementary File 1 Translated Survey Adherence to Caries Prevention Guidelines

**Knowledge of and compliance with Advice on Caries Prevention (ACP) guideline prevention**

Thank you in advance for filling out our questionnaire. We would like to ask you the following:

- Please answer all questions,
- Remember that there are no wrong answers; We want to know what you think and what is important to you,
- Read through all the answer options before choosing your answer. Then, tick the chosen answer.

Explanation and conditions of the research:

- I have understood the information. I have also had the opportunity to ask questions. My questions have been answered.
- I have had enough time to decide to participate or not.
- I know that I am not obligated to participate and that I do not need to share the reason for not participating.
- I understand that I can always stop if I no longer wish to participate.

My parent/guardian and I agree to participate in this study.

Declaration of consent:

- Yes
- No

Participants characteristics

1. How long have you had your braces now?
   - 0 to less than 3 months
   - 3 to less than 6 months
   - 6 to less than 12 months
   - Longer than 12 months
2. What kind of toothbrush do you use?
   - Manual toothbrush
   - Electric toothbrush
   - Both a manual toothbrush and an electric toothbrush
3. Are you?
   - A boy
   - A girl
   - Other namely…
   - I would rather not disclose
4. How old are you?
   - 12
   - 13
   - 14
   - 15
   - 16
   - 17
   - Other
5. What education are you enrolled in
   - Primary school [Basisschool]
   - Lower secondary vocational education [Voorbereidend Middelbaar Beroepsonderwijs; VMBO]
   - Senior general secondary education [Hoger Algemeen Voortgezet Onderwijs; HAVO]
   - Secondary vocational education [Middelbaar Beroepsonderwijs; MBO]
   - Pre-university education [Voorbereidend Wetenschappelijk Onderwijs; VWO]
   - University of applied sciences [Hoger Beroepsonderwijs; HBO]
   - University [Wetenschappelijk Onderwijs; WO]
   - No (formal) education
   - Other namely…
6. What is the highest education your mother has completed? (don’t know, first ask the researcher)
   - Primary school [Basisschool]
   - Lower secondary vocational education [Voorbereidend Middelbaar Beroepsonderwijs; VMBO]
   - Senior general secondary education [Hoger Algemeen Voortgezet Onderwijs; HAVO]
   - Secondary vocational education [Middelbaar Beroepsonderwijs; MBO]
   - Pre-university education [Voorbereidend Wetenschappelijk Onderwijs; VWO]
   - University of applied sciences [Hoger Beroepsonderwijs; HBO]
   - University [Wetenschappelijk Onderwijs; WO]
   - No (formal) education
   - I don’t know
   - Other namely…
7. What are the four digits of your postal code?
   - XXXX

Questions regarding the guideline

1. How often do you brush your teeth on average per day?
   - less than 1 time a day
   - 1 time a day
   - 2 times a day
   - 3 times a day
   - 4 times a day
   - 5 times a day
   - More than 5 times a day
2. How long do you brush your teeth on average at a time?
   - Less than 1 minute
   - 1 minute
   - 2 minutes
   - 3 minutes
   - 4 minutes
   - More than 4 minutes
   - I don’t know
   - Other, namely…
3. Do you use the products below? If so, which ones? (multiple answers possible)
   - Toothbrush
   - Interdental brush
   - Toothpicks
   - Dental floss
   - Fluoride mouthwash
   - Other, namely…
4. What kind of toothpaste do you use?
   - Toothpaste with fluoride
   - Fluoride-free toothpaste
   - Both toothpastes (with and without fluoride)
   - I don’t know
5. Have you been visiting your dentist in the past year for a checkup?
   - Yes
   - No
   - I don’t know

Explanation of question 6:

We would like to know more about your feeding moments. All moments when you eat or drink count as a feeding moment, except drinking water, coffee and tea without sugar. Eating and drinking at the same time counts as one feeding moment. If there is more than 30 minutes between two moments that you eat or drink something, it counts as the next feeding moment.

If it's not entirely clear, you can take a look at the example below.

EXAMPLE: Rachid has breakfast at 8 a.m. with two sandwiches and a glass of milk (1), at 10 a.m. he eats an apple (2) and at 11 a.m. he drinks a glass of water (no feeding moment). Then, at 1 p.m., he eats his salad for lunch with a glass of orange juice (3), after which he eats a cookie at 3 p.m. (4). At 5 p.m. he eats fried rice for dinner (5) and at 6 p.m. he has a bowl of custard (6). In the evening he has a glass of coke at 7 p.m. (7). Rachid has a total of 7 feeding moments.

*The numbers between brackets (1) represent the feeding moments.

Is there anything you do not understand? Please ask the researcher.

1. How often do you eat or drink something on average in a day?
   - Less than 4 times
   - 4-5 times
   - 6-7 times
   - 8-9 times
   - 10-11 times
   - More than 11 times
